# Leukocyte Mitochondrial DNA Copy Number and Risk of Thyroid Cancer: A Two-Stage Case-Control Study

**DOI:** 10.3389/fendo.2019.00421

**Published:** 2019-07-02

**Authors:** Jian Zheng, Ning-hua Cui, Shuai Zhang, Xue-bin Wang, Liang Ming

**Affiliations:** ^1^Department of Thyroid Surgery, The First Affiliated Hospital of Zhengzhou University, Zhengzhou, China; ^2^Zhengzhou Key Laboratory of Children's Infection and Immunity, Children's Hospital Affiliated to Zhengzhou University, Zhengzhou, China; ^3^Center for Gene Diagnosis, Zhongnan Hospital of Wuhan University, Wuhan, China; ^4^Department of Clinical Laboratory, the First Affiliated Hospital of Zhengzhou University, Zhengzhou, China

**Keywords:** mitochondrial DNA copy number, thyroid cancer, oxidative DNA damage, effect modification by BMI status, two-stage case-control study

## Abstract

**Background:** Mitochondrial DNA copy number (mtDNA-CN) may contribute to the development of various cancer types in a tumor-specific manner. However, little is known about whether leukocyte mtDNA content confers susceptibility to thyroid cancer (TC). This study aimed to investigate the associations of leukocyte mtDNA-CN with the risk and clinicopathological features of TC in a Chinese population.

**Methods:** In this two-stage case-control study with a total of 402 TC patients and 406 controls, leukocyte mtDNA-CN content was measured with a quantitative PCR method. In a subset of 100 cases and 100 controls, levels of leukocyte 8-hydroxy-2′-deoxyguanosine (8-OHdG) and plasma malondialdehyde, as two biomarkers for oxidative stress, were determined by ELISA and colorimetric kits, respectively.

**Results:** In a combined analysis of discovery and validation sets, high mtDNA-CN content was positively associated with increased TC risk, after adjusting for confounders (OR for per SD increment: 1.43; 95%CI, 1.23–1.66; *P* < 0.001; OR for tertile 3 vs. tertile 1: 2.10; 95%CI, 1.48–3.00; *P*_trend_ < 0.001). This linear dose-response relationship was more pronounced in subtype analyses for papillary and follicular thyroid carcinoma (*P* < 0.001 for all), as well as in subgroup analyses for subjects with overweight and obesity (*P*_interaction_ = 0.015). In TC patient, we observed the positive correlations of mtDNA-CN with advanced TNM stage (*P* = 0.006) and the presence of lymph node metastasis (*P* = 0.012). Leukocyte mtDNA-CN content was also identified to increase with the levels of leukocyte 8-OHdG (*P* < 0.001), a biomarker for oxidative DNA damage.

**Conclusion:** Our data suggest that the increase in leukocyte mtDNA-CN content may correlate with oxidative DNA damage, and serve as an independent risk factor for TC.

## Introduction

Thyroid cancer (TC) is the most common endocrine malignancy worldwide, with an estimated 298,000 new cases and 42,900 deaths annually (1, 2). Depending on the distinct patterns of clinical and biologic characteristics, TC is mainly classified into five histological subtypes: papillary thyroid carcinoma (PTC), follicular thyroid cancer (FTC), medullary thyroid carcinoma (MTC), poorly differentiated thyroid carcinoma (PDTC), and anaplastic thyroid carcinoma (ATC) (3). One vital pathway for the development of TC is oxidative stress, which may form oxidative lesions of DNA, cause genomic instability, and consequently initiate tumourigenesis in thyroid cells (4).

Mitochondria, as cellular energy-generating organelles in eukaryotic cells, are mainly responsible for the synthesis of adenosine triphosphate and modulation of oxidative stress (5). With advancing age, mitochondria may produce increasing reactive oxygen species (ROS), which in turn induce mitochondrial DNA (mtDNA) damage and mutations, impair mitochondrial functions, and consequently cause the oncocytic phenotype during tumorigenesis in thyroid (6–8). Notably, mitochondrial DNA copy number (mtDNA-CN), as a surrogate marker of mitochondrial function (9), has been reported to correlate with various cancer types in a tumor-specific manner (10), with some studies revealing high mtDNA-CN in leukocytes associated with the increased risk of lymphoma and breast cancer, and others suggesting the protection of increased mtDNA-CN against hepatic carcinoma and skeleton cancer (11, 12). However, to date, the association of leukocyte mtDNA-CN with susceptibility to TC was barely investigated.

Hence, we conducted a two-stage case-control analysis to assess the associations of leukocyte mtDNA-CN with the risk of TC, the clinicopathological features of TC, and oxidative DNA damage in the Chinese Han population.

## Materials and Methods

### Study Population

This study used a two-stage case-control design, involving a total of 402 TC patients and 406 controls. The discovery set, composed of 152 cases and 151 controls, was collected from Zhongnan Hospital of Wuhan University at Hubei Province (central China) between June 2017 and February 2019. The validation set of 250 cases and 255 controls was enrolled from The First Affiliated Hospital of Zhengzhou University at Henan Province (northern China) between April 2018 and March 2019. The inclusion criteria for the cases were histology-confirmed primary TC (by two independent pathologists), without any surgical management and radioiodine therapy prior to sample collection. The control group was consisted of healthy volunteers who received routine physical examinations, and frequency-matched to the cases on age (±5 years), sex, and living area. For all participants, we recorded the demographic data previously associated with TC, including smoking status (13), alcohol drinking status (14), body mass index (BMI) (15), and history of diabetes mellitus (DM) (16) (Supplementary Materials and Methods). For TC patients, we also documented the clinicopathological data of TC, including histological subtypes, TNM stage, tumor size, multifocality, status of lymph node metastasis, and levels of thyroid stimulating hormone (TSH). All individuals were self-reported Han Chinese. The study protocol of two sets was approved by local ethics committees and participants signed written informed consents accordingly.

### Measurement of Leukocyte mtDNA-CN

Genomic DNA of peripheral blood leukocytes was isolated using the QIAamp DNA mini kits (Qiagen, Dusseldorf, Germany). Relative mtDNA-CN was measured by a quantitative PCR (qPCR) method, as described by our previous study (17). Briefly, the copy number ratio of mitochondrial *ND1* gene to a single-copy nuclear gene (*HGB*) was quantified for each sample using a seven-point standard curve. The standard curve for each batch was generated by the 2-fold serial dilutions (range: 0.3125–20 ng/μl) of a reference DNA sample. Then, the ratio of each sample was normalized with a calibrator DNA sample using the 2^−ΔΔCq^ equation, and transformed into the relative quantity of mtDNA-CN. The calibrator was a mixed DNA sample pooled from 10 randomly selected controls. qPCR reactions for each sample were assayed in triplicate on a LC480 SYBR Green System (Roche, Indianapolis, IN, USA). The primer sequences for the mitochondrial *ND1* gene were: 5′-CCC TAA AAC CCG CCA CAT CT-3′ (forward); 5′-GAG CGA TGG TGA GAG CTA AGGT-3′ (reverse). The primers for the *HGB* reference gene were: 5′-GCT TCT GAC ACA ACT GTG TTC ACT AGC-3′ (forward); 5′-CAC CAA CTT CAT CCA CGT TCA CC-3′ (reverse). Each PCR reaction was performed in a final volume of 10 μL reaction mixture containing 5 μL of 1 × SYBR green master Mix, 10 nM of each primer, and 4 ng of genomic DNA. The thermal cycling conditions for both primers were 95°C for 10 min, followed by 40 cycles of 95°C for 15 s, 60°C for 1 min.

The steps of quality control were as follows. First, the *R*^2^ for each standard curve should be >0.99, with standard deviations (SDs) of the Cq values of <0.25 and qPCR efficiencies of >1.95. Otherwise, the test was repeated. Second, to control position effects, qPCR reactions for *ND1* and *HGB* were invariably conducted on separate 384-well plates with the same samples in the same wells. Third, four quality controls were inserted into each plate to calculate inter-batch variation. The averaged CV across all batches was 3.5% for the mtDNA-CN assays. Finally, qPCR procedures were carried out by investigators who were blinded to clinical data and disease status.

### Detection of Oxidative Stress Biomarkers

For grouping a subset of participants receiving the measurement of oxidative parameters, we first randomly selected 100 cases from two cohorts, and then matched them in a 1:1 ratio with 100 age- and sex-matched controls using a propensity score matching method. Leukocyte 8-hydroxy-2′-deoxyguanosine (8-OHdG) and plasma malondialdehyde (MDA), as biomarkers for oxidative DNA damage and lipid peroxidation, respectively (18, 19), were detected in the subset. For detection of leukocyte 8-OHdG levels, genomic DNA of leukocytes was first digested with 0.2 U/μg of P1 nuclease, 0.4 U/μg of phosphodiesterase, and 0.04 U/μg of alkaline phosphatase to yield free deoxyguanosine (dG) (20). Then, the quantity of dG was determined at 254 nm; the quantity of 8-OHdG was detected by a competitive ELSIA kit (Trevigen Inc, Gaithersburg, MD, USA) following the manufacturer's instructions. Levels of 8-OHdG were finally calculated as the ratio of 8-OHdG/10^5^ dG. Plasma MDA levels were measured with the thiobarbituric acid reactive substances assay, as described by our previous reports (17) (Supplementary Materials and Methods).

### Statistical Analyses

The differences in demographics between cases and controls were analyzed by the student's *t*-test for continuous variables, and by the Pearson χ^2^ test for categorical variables. We used z transformation to standardize the distribution of mtDNA-CN data across two cohorts. The associations of mtDNA-CN *z* scores with TC risk were assessed by logistic regression models with and without adjustment for age, sex, BMI, smoking status, alcohol drinking status, and history of DM. In regression models, mtDNA-CN was first investigated as a continuous variable, and then as an ordinary variable by using the tertile cutpoints in controls for variable distributions. We also constructed a restricted cubic spline (21) to visualize the shape of the associations between mtDNA-CN and TC risk. In stratified analyses, the χ^2^-based *Q* test was applied to evaluate the effect modification by lifestyle risk factors of TC. We used multivariable linear regression to assess the correlations of oxidative stress biomarkers with TC risk and mtDNA-CN after adjusting for covariates. All above analyses were conducted with SPSS 17.0 software (SPSS Inc., Chicago, IL, USA). A statistical power was calculated by PS3.0 software (Vanderbilt University, Nashville, TN, USA).

## Results

### Demographics of Study Populations

In both discovery and validation sets (Table 1), patients with TC had a higher level of BMI and a higher frequency of DM than controls. There were no significant differences in the distributions of age, sex, smoking status, and alcohol drinking status between cases and controls. We also did not observe significant differences in demographics between the discovery and validation sets in either the case group or the control group (Table S1), suggesting that the data of two sets were comparable.

**Table 1:** Clinical characteristics of participants in our study.

**Variables^a^**	**Discovery set**			**Validation set**			**Combined set**		
	**TC (*N* = 152)**	**Controls (*N* = 151)**	***P*^a^**	**TC (*N* = 250)**	**Controls (*N* = 255)**	***P*^a^**	**TC (*N* = 402)**	**Controls (*N* = 406)**	***P*^a^**
Age, years	53.0 ± 8.3	51.5 ± 8.9	0.136	52.2 ± 7.5	51.7 ± 8.8	0.425	52.5 ± 7.8	51.6 ± 8.8	0.117
Female, *n* (%)	95 (62.5)	88 (58.3)	0.452	153 (61.2)	161 (63.1)	0.654	248 (61.7)	249 (61.3)	0.916
Smoking, *n* (%)	41 (27.0)	51 (33.8)	0.198	64 (25.6)	78 (30.6)	0.213	105 (28.1)	129 (31.8)	0.076
Alcohol drinking, *n* (%)	38 (25.0)	52 (34.4)	0.072	66 (26.4)	59 (23.1)	0.396	104 (25.9)	111 (27.3)	0.637
History of DM, n (%)	53 (34.9)	33 (21.9)	0.012	91 (36.4)	59 (23.1)	0.001	144 (35.8)	92 (22.7)	< 0.001
BMI, kg/m^2^	25.2 ± 3.6	24.4 ± 2.2	0.028	25.1 ± 3.5	24.4 ± 2.5	0.016	25.1 ± 3.5	24.4 ± 2.4	0.001
**TC subtypes**, ***n*** **(%)**									
PTC	117 (77.0)			193 (77.2)			310 (77.1)		
FTC	25 (16.4)			47 (18.8)			72 (17.9)		
MTC	10 (6.6)			2 (0.8)			12 (3.0)		
PDTC and ATC	0 (0)			8 (3.2)			8 (2.0)		

*PTC, papillary thyroid carcinoma; FTC, follicular thyroid cancer; MTC, medullary thyroid carcinoma; PDTC, poorly differentiated thyroid carcinoma; ATC, anaplastic thyroid carcinoma*.

a *Continuous variables were expressed as mean ± SD, and compared by the Student's t-test. Categorical variables were expressed as frequency counts, and compared by the Pearson χ^2^ test*.

### Associations of Leukocyte mtDNA-CN *z* Scores With TC Risk

**Figure 1:**
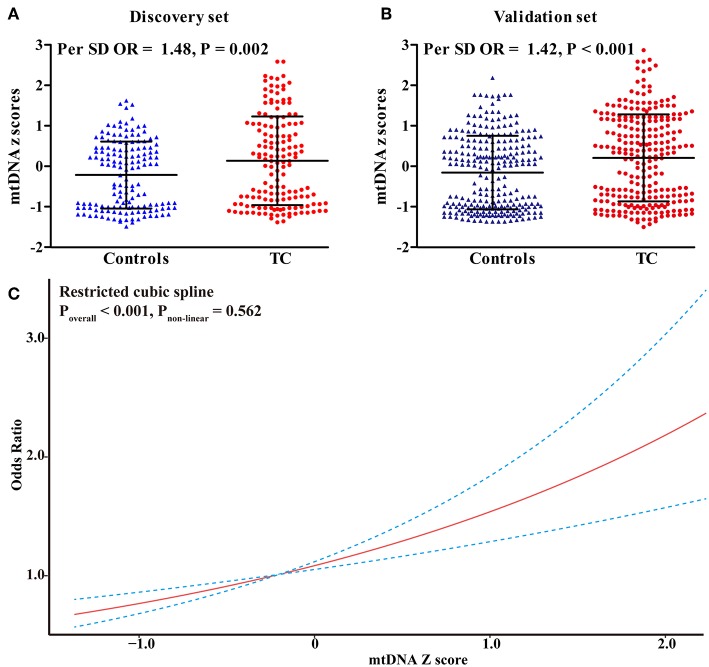
Risk assessment of leukocyte mtDNA-CN *z* scores in CC. **(A)** The difference in leukocyte mtDNA-CN *z* scores between cases and controls in the discovery set; **(B)** The difference in leukocyte mtDNA-CN *z* scores between cases and controls in the validation set; **(C)** Restricted cubic spline for assessing the shape of the association between leukocyte mtDNA-CN *z* scores and TC risk in the combined set. In, restricted cubic spline, four knots were set at 5th, 35th, 65th, and 95th percentiles of mtDNA-CN *z* scores. The red line represented the ORs corresponding to the different *z* scores of mtDNA-CN. The blue dotted line represented the 95%CIs of the ORs. ORs and *P*-values adjusted for age, sex, BMI, smoking habit, alcohol drinking habit, and history of DM.

In the discovery set from Hubei Province, central China, mtDNA-CN *z* scores in TC patients were significantly higher than those in controls (Figure 1A). After adjusting for covariates, each 1 standard deviation (SD) increase in mtDNA-CN *z* scores was associated with a 48% (95%CI, 1.15–1.91; *P* = 0.002) increased risk of TC. This association was further replicated in a larger cohort from Henan Province, northern China (Figure 1B). In this validation set, the ORs for the association of continuous mtDNA-CN *z* scores with TC risk were 1.44 (95%CI, 1.20–1.72; *P* < 0.001) in the crude model, and 1.42 (95%CI, 1.18–1.71; *P* < 0.001) in the multivariable-adjusted model. Then, to fully increase statistical power, we performed a meta-analysis of two sets, which identified a 43% (95%CI, 1.23–1.66; *P* < 0.001) increase in TC risk per 1-SD increment in mtDNA-CN *z* scores (Table 2). Assuming an OR of 1.43, the entire population could provide a statistical power of 81.4% (α = 0.05) to clarify the association. When investigating mtDNA-CN as an ordinary variable, the risk of developing TC increased significantly across the tertiles of mtDNA-CN (*P*_trend_ < 0.001). Of note, participants in the top tertile of mtDNA-CN had a 2.10-fold (95%CI, 1.48–3.00; *P* < 0.001) increased risk of TC, compared with those in the bottom tertile of mtDNA-CN. Consistent with the results of logistic regression, the restricted cubic spline showed that the odds of developing TC increased with mtDNA-CN *z* scores (*P*_overall_ < 0.001, *P*_non−linear_ = 0.562, Figure 1C), suggesting a linear dose-response association between mtDNA-CN and TC risk.

**Table 2:** Associations of leukocyte mtDNA-CN with TC risk in discovery, validation, and their combined sets.

**Variables**	**Cases, *N***	**Controls, *N***	**Without adjustment**		**With adjustment^a^**	
			**OR (95%CI)**	***p***	**OR (95%CI)**	***p***
**DISCOVERY STAGE**						
Each 1-SD increase	152	151	1.45 (1.14–1.84)	0.002	1.48 (1.15–1.91)	0.002
**REPLICATION STAGE**						
Each 1-SD increase	250	255	1.44 (1.20–1.72)	<0.001	1.42 (1.18–1.71)	<0.001
**COMBINED COHORT**						
Each 1-SD increase	402	406	1.44 (1.25–1.66)	<0.001	1.43 (1.23–1.66)	<0.001
**BY TERTILES IN CONTROLS**						
T1 (≤ −0.93)	93 (23.1)	138 (34.0)	Reference		Reference	
T2 (−0.93–0.30)	118 (29.4)	135 (33.2)	1.30 (0.90–1.86)	0.158	1.28 (0.89–1.86)	0.183
T3 (>0.30)	191 (47.5)	133 (32.8)	2.13 (1.51–3.01)	<0.001	2.10 (1.48–3.00)	<0.001
*P* trend			<0.001		<0.001	

a *Adjusted for age, sex, BMI, smoking habit, alcohol drinking habit, and history of DM*.

### Stratified Analyses for Observing the Effect Modification by Lifestyle Risk Factors of TC

**Figure 2:**
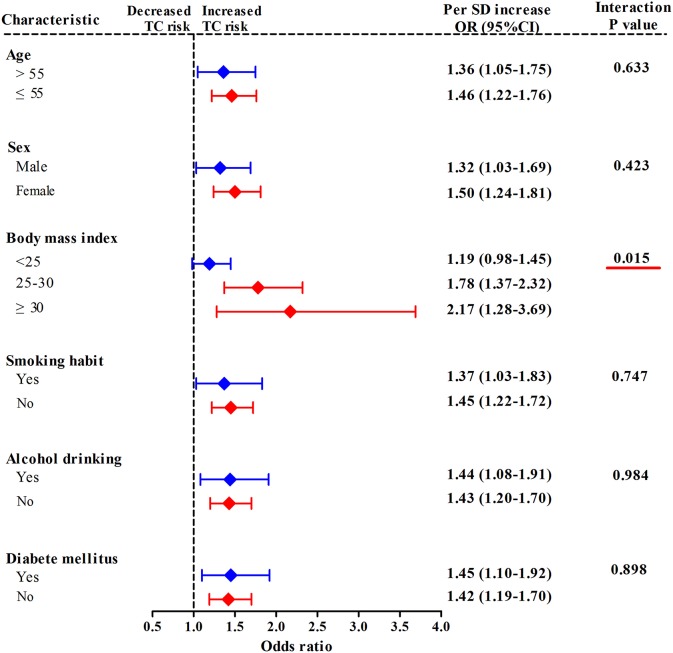
Stratified analyses for assessing the effect modification by lifestyle risk factors of TC for the association between leukocyte mtDNA-CN *z* scores and TC risk. ORs and 95% CIs adjusted for age, sex, BMI, smoking habit, alcohol drinking habit, and history of DM. *P*_interaction_ were obtained from the χ^2^-based Q test. Red underlines represented *P*_interaction_ < 0.005 for effect modification analysis.

In a stratified analysis by BMI status (Figure 2), with each 1-SD increase in mtDNA-CN *z* scores, the adjusted odds of developing TC increased by up to 2.17-fold (95%CI, 1.28–3.69; *P* = 0.004) in participants with obesity (BMI ≥ 30 kg/m^2^), by 1.78-fold (95%CI, 1.37–2.32; *P* < 0.001) in subjects with overweight (25 kg/m^2^ ≤ BMI < 30 kg/m^2^), and only by 1.19-fold (95%CI, 0.98–1.45; *P* = 0.084) in those with BMI < 25 kg/m^2^, suggesting an effect modification by BMI status for the association between mtDNA-CN and TC risk (*P*_interaction_ = 0.015). When participants were stratified by age, sex, smoking status, alcohol drinking status, and history DM, each 1-SD increase in mtDNA-CN consistently conferred an increased risk of TC in all subgroups, with no effect modification observed (*P*_interaction_ > 0.05).

### Associations of Leukocyte mtDNA-CN With Different Histological Subtypes of TC

We next analyzed the associations of leukocyte of mtDNA-CN with different subtypes of TC in the combined cohort. In the multivariable-adjusted models (Table 3), a 1-SD increase in mtDNA-CN *z* scores correlated with a 1.43-fold increased (95%CI, 1.22–1.68; *P* < 0.001) risk of PTC and a 1.66-fold increased (95%CI, 1.26–2.18; *P* < 0.001) risk of FTC, but not with the risk of MTC as well as PDTC and ATC, suggesting a stronger effect of mtDNA-CN on differentiated TC than on other subtypes. Particularly, in a comparison of top vs. bottom tertile of mtDNA-CN *z* scores, the adjusted ORs reached 2.09 (95%CI, 1.43–3.06; *P* < 0.001) for PTC risk, and 2.79 (95%CI, 1.41–5.51; *P* = 0.003) for FTC risk.

**Table 3:** Associations of leukocyte mtDNA-CN with subtypes of TC risk in the combined sets.

**mtDNA-CN**	**Control**	**PTC**			**FTC**			**MTC**		**PDTC and ATC**	
	***N* (%)**	***N* (%)**	**OR (95%CI)^a^**	***P*^a^**	***N* (%)**	**OR (95%CI)^a^**	***P*^a^**	**OR (95%CI)^a^**	***P*^a^**	**OR (95%CI)^a^**	***P*^a^**
Each 1-SD increase	406	310	**1.43 (1.22–1.68)**	**<0.001**	72	**1.66 (1.26–2.18)**	**<0.001**	1.54 (0.47–5.02)	0.477	1.10 (0.25–4.81)	0.903
**BY TERTILES IN CONTROLS**											
T1 (≤ −0.93)	138 (34.0)	72 (23.3)	Reference		14 (19.4)	Reference		Reference		Reference	
T2 (−0.93–0.30)	135 (33.2)	92 (29.7)	1.29 (0.86–1.91)	0.215	21 (29.2)	1.54 (0.74–3.20)	0.252	0.45 (0.08–2.42)	0.352	1.48 (0.24–9.10)	0.672
T3 (>0.30)	133 (32.8)	146 (47.1)	**2.09 (1.43–3.06)**	**<0.001**	37 (51.4)	**2.79 (1.41–5.51)**	**0.003**	1.11 (0.30–4.04)	0.878	1.53 (0.25–9.53)	0.647
*p* trend				**<0.001**			**0.008**		0.558		0.885

a *Adjusted for age, sex, BMI, smoking habit, alcohol drinking habit, and history of DM. The main purpose of the bold values is to more differentially present the data above and below the bold line*.

### Associations of Leukocyte mtDNA-CN With Clinicopathological Features of TC

**Figure 3:**
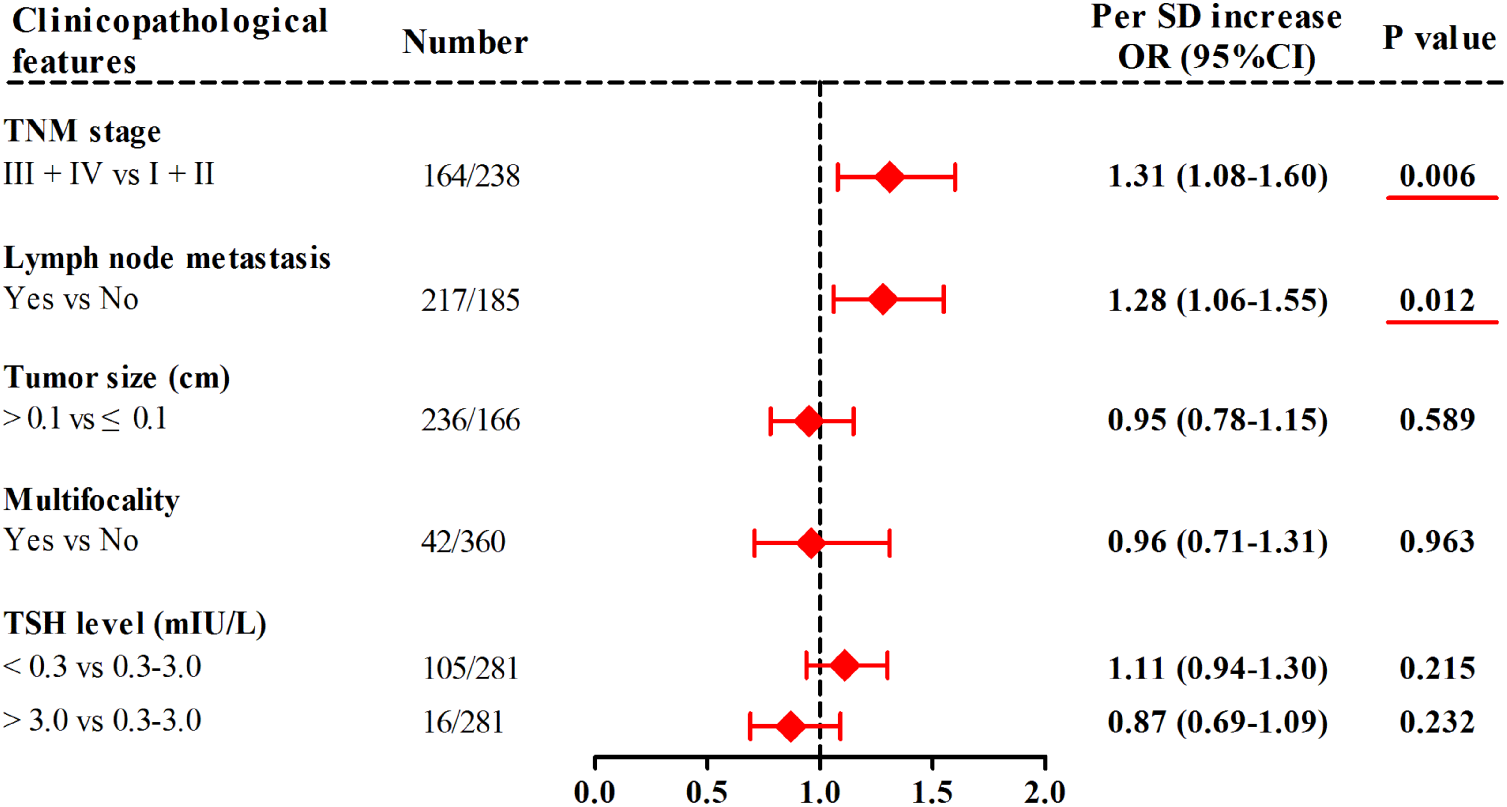
Associations of leukocyte mtDNA-CN *z* scores with clinicopathological features of TC. ORs and *P*-values adjusted for age, sex, BMI, smoking habit, alcohol drinking habit, and history of DM. Red underlines represented *P*-values < 0.05.

In TC patients from the combined cohort (Figure 3), with 1-SD increment in mtDNA *z*-scores, the adjusted odds of having advanced TNM stage and lymph node metastasis increased by 31% (95%CI, 1.08–1.60; *P* = 0.006) and 28% (95%CI, 1.06–1.55; *P* = 0.012), respectively. Otherwise, we did not find significant associations of leukocyte mtDNA-CN with tumor size, multifocality, and TSH levels.

### Associations of Oxidative Stress Biomarkers With TC Risk and Leukocyte mtDNA-CN

**Figure 4:**
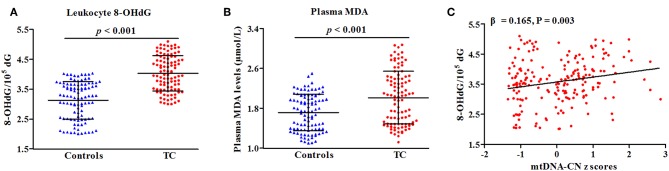
Correlations of oxidative parameters with TC risk **(A,B)** and mtDNA-CN *z* scores **(C)**. β and *P*-values adjusted for age, sex, BMI, smoking habit, alcohol drinking habit, and history of DM.

In a subset of 100 cases and 100 controls (Table S2), levels of leukocyte 8-OHdG and plasma MDA were quantified to reflect the overall status of oxidative stress. As presented in Figure 3, patients with TC had increased levels of leukocyte 8-OHdG and plasma MDA as compared with controls (*P* < 0.001 for all, Figures 4A,B). There was a positive correlation of leukocyte mtDNA-CN with 8-OHdG levels (β = 0.165, *P* = 0.003, Figure 4C). Of note, a stratified analysis by BMI status showed that the positive association between 8-OHdG and mtDNA-CN was only significant in subjects with BMI ≥ 25 kg/m^2^ (β = 0.358, *P* < 0.001), but not in those with BMI < 25 kg/m^2^ (β = −0.031, *P* = 0.666). No significant association was found between plasma MDA and leukocyte mtDNA-CN.

## Discussion

MtDNA-CN, as a biomarker of mitochondrial function, has been widely reported to increase with the extent of oxidative stress in senescent or cancer cells (22, 23). In experimental studies, the ROS-induced increase in mtDNA molecules was considered as a consequence of compensatory response to the inhibition of mitochondrial respiratory function caused by persistent accumulation of mtDNA mutations (8, 24). In turn, the increased mitochondrial biogenesis would generate much more ROS, which formed a vicious cycle to induce more severe oxidative damage and tumorigenesis (25). However, in recent meta-analyses of observational studies, leukocyte mtDNA-CN content was suggested to correlate positively with the risk of lymphoma and breast cancer, but negatively with hepatic carcinoma and skeleton cancer (11, 12), suggesting that alterations in leukocyte mtDNA-CN might be regulated in a tumor-specific manner during carcinogenesis (10). Specific to TC, previous studies provided clues that mtDNA-CN content, measured in thyroid tissues, might predict the occurrence of large scale mtDNA deletions (26) and oncocytic phenotype in TC (27). However, little is known about whether leukocyte mtDNA, as a mitochondrial marker that is easier to detect, contributes to TC risk. In light of this, we designed a two-stage case control study aiming to investigate the association between leukocyte mtDNA-CN and TC. Collectively, we found a linear dose-response relationship between high leukocyte mtDNA-CN and increased TC risk in the Chinese population. Then, in TC patients, leukocyte mtDNA-CN was further identified to correlate with advanced TNM stage and the presence of lymph node metastasis. Finally, we observed a positive correlation of leukocyte mtDNA-CN with 8-OHdG, a biomarker for oxidative DNA damage. All these results together suggest that high leukocyte mtDNA-CN may be an independent risk factor for TC.

In the present study, we also observed a stronger effect of high leukocyte mtDNA-CN content on PTC and FTC than on other histological subtypes. Several lines of evidence may help to interpret this result. First, PTC and FTC, as the two most common subtypes arising from differentiated follicular cells (3), accounted for more than 85% of TC in our study. The relatively large sample size might be beneficial to show the significant association of mtDNA-CN with PTC and FTC. Second, PTC, as a tumor type with low densities of mutations, have the most common driver mutation termed BRAF V600E (28), which has been reported to down-regulate the expression of gene clustering in the mitochondrial electron transport chain pathway (29), induce mitochondrial localization (30), and consequently inhibit mitochondrial respiration and metabolism in TC (31). So, high leukocyte mtDNA-CN content may serve as a compensatory response to mitochondrial dysfunction (24), thus greatly increasing the risk of PTC. This notion was also supported by a recent observation that mtDNA-CN in PTC tissues was nearly 4-times higher than in normal thyroid tissues (8). Third, although there was no direct evidence supporting the casual roles of mtDNA-CN content in the pathogenesis of FTC, a recent study by parallel analysis of mtDNA and nuclear genomes identified a marked increase in mitochondria as the genetic driver in a variant of FTC, i.e., Hürthle cell carcinoma of the thyroid (32), implying a possible link between increasing mtDNA-CN content and pathogenesis of FTC.

In this study, a stratified analysis by BMI status showed that with each 1-SD increase in mtDNA-CN, the adjusted odds of developing TC increased by 1.78-fold in subjects with overweight and by up to 2.17-fold in participants with obesity. Adiposity, as a well-known risk factor for TC, has been reported as a modulator for insulin resistance, chronic inflammation, and increase in TSH secretion, all of which were crucial contributors to TC (33–35). Moreover, besides the direct effect of adiposity on the development of TC, accumulated evidence has suggested the casual roles of impaired mitochondrial biogenesis in acquired obesity (36), as well as the induction of uncontrolled oxidative stress by excess of adiposity (37), implying a cumulative effect of changes in mtDNA-CN and adiposity on modulating TC risk. The above findings, combined with the current observation that leukocyte mtDNA-CN showed a strongly positive correlation with leukocyte 8-OHdG levels in participants with BMI ≥ 25 kg/m^2^, suggest that the interaction between increment of mtDNA-CN and adiposity may greatly aggravate oxidative DNA damage, and increase TC risk.

This study had some limitations. First, although this is a two-stage case-control study enrolled participants from two districts of China, we could not completely solve the reverse-causation problem due to the nature flaw of a retrospective design. Second, the data of mtDNA-CN in thyroid tissues were not acquired, so we could not assess the tissue-specific association between mtDNA-CN and TC risk. Third, the present study only found a positive correlation between leukocyte mtDNA-CN and 8-OHdG levels. However, the exact ROS-inducing agents that cause oxidative DNA damage were not identified. Finally, although we collected several modifiable risk factors for TC, other confounders, especially the genetic data on BRAF V600E mutation, were not recorded for further adjustment.

In summary, the present study, for the first time, suggests that the increase in leukocyte mtDNA-CN content may correlate with a biomarker for oxidative DNA damage (8-OHdG level), and serve as an independent risk factor for TC.

## Data Availability

The raw data supporting the conclusions of this manuscript will be made available by the authors, without undue reservation, to any qualified researcher.

## Ethics Statement

This study was carried out in accordance with the Principles of the Declaration of Helsinki with written informed consent from all subjects. The protocol was approved by the ethics committees of First Affiliated Hospital of Zhengzhou University and Zhongnan Hospital of Wuhan University.

## Author Contributions

XW and JZ conceived and designed the experiments. NC and XW conducted the experiments. SZ and XW collected clinical data and samples. XW and JZ conducted statistical analyses. LM wrote the manuscript.

### Conflict of Interest Statement

The authors declare that the research was conducted in the absence of any commercial or financial relationships that could be construed as a potential conflict of interest.
