# Supplementary material for: Leukocyte Mitochondrial DNA Copy Number and Risk of Thyroid Cancer: A Two-Stage Case-Control Study
Source: Front Endocrinol (Lausanne). 2019 Jul 2;10:421. doi: 10.3389/fendo.2019.00421 (PMC6614343; doi:10.3389/fendo.2019.00421)
Supplement: Supplementary file 1 [file Data_Sheet_1.doc]

**Supplementary Files**

**Leukocyte mitochondrial DNA copy number and risk of thyroid cancer: A two-stage case-control study**

**Running title: mtDNA copy number and thyroid cancer**

Jian Zheng ^1^, Ning-hua Cui ^2^, Shuai Zhang ^3^, Xue-bin Wang ^4*^, and Liang Ming ^4*^

^1^ Department of Thyroid Surgery, The First Affiliated Hospital of Zhengzhou University, Zhengzhou, Henan, China

^2^ Zhengzhou Key Laboratory of Children's Infection and Immunity, Children's Hospital Afliated to Zhengzhou University, Henan, China

^3^ Center for Gene Diagnosis, Zhongnan Hospital of Wuhan University, Wuhan, Hubei, China

^4^ Department of Clinical Laboratory, The First Affiliated Hospital of Zhengzhou University, Zhengzhou, Henan, China

**CORRESPONDENCE**

Liang Ming

Email: mingliangjyk2011@163.com;

Xue-bin Wang

Email: xbwang2017@163.com;

**Supplementary Materials and Methods**

**Definition of lifestyle risk factors for TC**

Individuals who smoked ≥ 100 cigarettes in their lifetime were defined as “smokers”, which included ever smokers and current smokers. An ever smoker was a person who had quit smoking at least 1 year prior to interview. Subjects with alcohol consumption at least once a week for ≥ 1 year were defined as “alcohol drinkers”. One drink was defined as 375 ml of beer (13.6 g of ethanol), 118 ml of wine (11.7 g of ethanol), or 30 ml of western or Chinese hard liquor (10.9 g of ethanol). The BMI cutpoints for defining overweight and obesity were 25.0-29.9 kg/m^2^ and ≥ 30 kg/m^2^, respectively, based on the Obesity Society with the ACC/AHA Task Force on Practice Guidelines. Diabetes mellitus (T2DM) was defined as ongoing therapy for diabetes or fasting plasma glucose (FPG) levels of ≥ 7.0 mmol/L, or plasma glucose levels of ≥ 11.1 mmol/L, or a 2-h plasma glucose level of ≥ 11.1 mmol/L during an oral glucose tolerance test.

**Measurement of plasma MDA levels**

For quantification of MDA, 100 μL of plasma samples were freshly isolated by centrifugation (at 2000g for 10 min at 4 ℃), and incubated with 100 μL of 10% trichloroacetic acid to deproteinize. After centrifugation (at 13000 g for 5 min), 100 μL of supernatant was added to 100 μL of thiobarbituric acid, and heated at 95 ℃ for 10 min. Then, the sample was cooled, and mixed with 200 μL of n-butanol. After centrifugation (at 13000 g for 3 min), 100 μL of supernatant was collected to quantify the levels of MDA at 532 nm.

| **Table S1:** Comparisons of demographics between discovery set and validation set | | | | | | | | |
| --- | --- | --- | --- | --- | --- | --- | --- | --- |
| Variables ^a^ | Cases | | |  | Controls | | |  |
|  | Discovery  (N = 152) | Validation  (N = 250) | P ^a^ |  | Discovery  (N = 151) | Validation  (N = 250) | P ^a^ |  |
| Age, years | 53.0 ± 8.3 | 52.2 ± 7.5 | 0.356 |  | 51.5 ± 8.9 | 51.7 ± 8.8 | 0.860 |  |
| Female, n (%) | 95 (62.5) | 153 (61.2) | 0.795 |  | 88 (58.3) | 161 (63.1) | 0.331 |  |
| Smoking, n (%) | 41 (27.0) | 64 (25.6) | 0.761 |  | 51 (33.8) | 78 (30.6) | 0.505 |  |
| Alcohol drinking, n (%) | 38 (25.0) | 66 (26.4) | 0.756 |  | 52 (34.4) | 59 (23.1) | 0.054 |  |
| History of DM, n (%) | 53 (34.9) | 91 (36.4) | 0.756 |  | 33 (21.9) | 59 (23.1) | 0.765 |  |
| BMI, kg/m^2^ | 25.2 ± 3.6 | 25.1 ± 3.5 | 0.831 |  | 24.4 ± 2.2 | 24.4 ± 2.5 | 0.981 |  |
| TC subtypes, n (%) |  |  |  |  |  |  |  |  |
| PTC | 117 (77.0) | 193 (77.2) | 0.951 |  |  |  |  |  |
| FTC | 25 (16.4) | 47 (18.8) |  |  |  |  |  |  |
| MTC | 10 (6.6) | 2 (0.8) |  |  |  |  |  |  |
| PDTC and ATC | 0 (0) | 8 (3.2) |  |  |  |  |  |  |
| mtDNA-CN z scores | 0.13 ± 1.10 | 0.21 ± 1.07 | 0.516 |  | -0.16 ± 0.90 | 0.22 ± 0.83 | 0.506 |  |
| PTC: papillary thyroid carcinoma; FTC: follicular thyroid cancer; MTC: medullary thyroid carcinoma; PDTC: poorly differentiated thyroid carcinoma; ATC: anaplastic thyroid carcinoma.  ^a^ Continuous variables were expressed as mean ± SD, and compared by the Student's t-test. Categorical variables were expressed as frequency counts, and compared by the Pearson χ^2^ test. | | | | | | | | |

| **Table S2:** Comparisons of demographics between subset cases and total cases, and between subset controls and total controls. | | | | | | | | |
| --- | --- | --- | --- | --- | --- | --- | --- | --- |
| Variables |  | Subset TC vs Total TC | | |  | Subset controls vs Total controls | | |
|  |  | Subset (n = 100) | Total (n = 402) | P ^a^ |  | Subset (n = 100) | Total (n = 406) | P ^a^ |
| Age, years |  | 52.9 ± 7.4 | 52.5 ± 7.8 | 0.630 |  | 51.1 ± 9.1 | 51.6 ± 8.8 | 0.610 |
| Female, n (%) |  | 60 (60.0) | 248 (61.7) | 0.756 |  | 64 (64.0) | 249 (61.3) | 0.622 |
| Smoking, n (%) |  | 28 (28.0) | 105 (26.1) | 0.703 |  | 29 (29.0) | 129 (31.8) | 0.592 |
| Alcohol drinking, n (%) |  | 28 (28.0) | 104 (25.9) | 0.665 |  | 26 (26.0) | 111 (27.3) | 0.787 |
| History of DM, n (%) |  | 35 (35.0) | 144 (35.8) | 0.878 |  | 25 (25.0) | 92 (22.7) | 0.619 |
| BMI, kg/m^2^ |  | 25.2 ± 3.5 | 25.1 ± 3.5 | 0.920 |  | 24.4 ± 2.5 | 24.4 ± 2.4 | 0.984 |
| TC subtypes, n (%) |  |  |  |  |  |  |  |  |
| PTC |  | 77 (77.0) | 310 (77.1) | 0.255 |  | 310 (77.1) |  |  |
| FTC |  | 17 (17.0) | 72 (17.9) |  |  | 72 (17.9) |  |  |
| MTC |  | 6 (6.0) | 12 (3.0) |  |  | 12 (3.0) |  |  |
| PDTC and ATC |  | 0 (0) | 8 (2.0) |  |  | 8 (2.0) |  |  |
| mtDNA, z scores |  | 0.21 ± 1.07 | 0.18 ± 1.08 | 0.807 |  | -0.15 ± 0.83 | -0.18 ± 0.88 | 0.808 |
| PTC: papillary thyroid carcinoma; FTC: follicular thyroid cancer; MTC: medullary thyroid carcinoma; PDTC: poorly differentiated thyroid carcinoma; ATC: anaplastic thyroid carcinoma.  ^a^ Continuous variables were expressed as mean ± SD, and compared by the Student's t-test. Categorical variables were expressed as frequency counts, and compared by the Pearson χ^2^ test. | | | | | | | | |
